# Supplementary material for: Recognizing IgA-class endomysial antibody equivalent binding patterns on monkey liver substrate through EfficientNet architectures and deep learning
Source: PeerJ. 2025 Oct 15;13:e20191. doi: 10.7717/peerj.20191 (PMC12535235; doi:10.7717/peerj.20191)
Supplement: Supplemental Information 1 [file peerj-13-20191-s001.pdf]

Supplementary Table 1
TTG Analysis Results Table

July 16, 2025

**Image**	**Anti TTG IgA ELISA (≻20 RU/mL positive)**	**Anti TTG result**	**Experts' Final Consideration**	**ML Prediction**
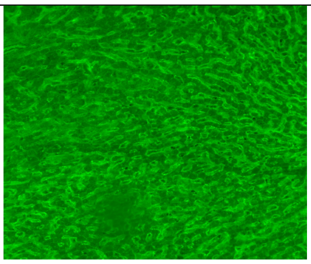	7,6	Negative	Heterogeneous staining, similar to +1 positive but Anti-TTG negative patient	Gray Zone
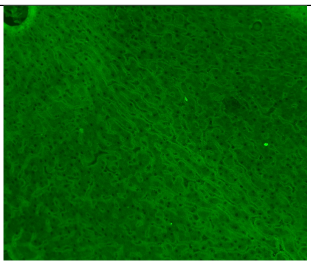	48,3	Positive	Heterogenous staining, neither +1 positive nor negative.	Gray Zone
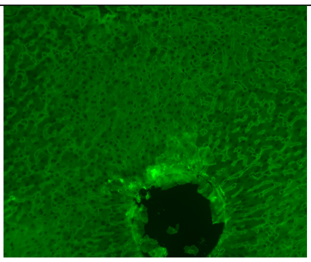	23,7	Positive	Heterogenous staining, neither +1 positive nor negative.	Gray Zone
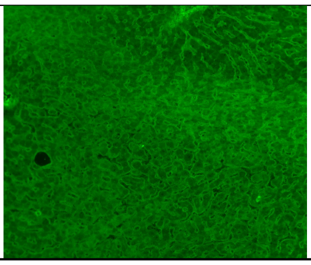	<2	Negative	Near to +1 positive, but Anti TTG negative	Gray Zone

Continued on next page

Image	Anti TTG IgA ELISA ($\succ$20 RU/mL positive)	Anti TTG result	Experts' Final Consideration	ML Prediction
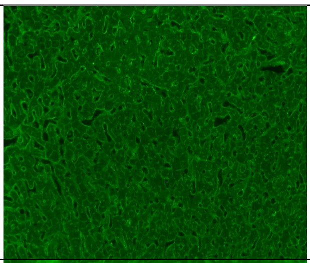	<2	Negative	Near to +1 positive, but Anti TTG negative	Weak Positive
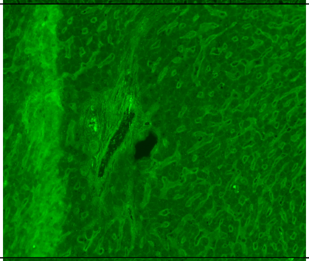	<2	Negative	Near to +1 positive, but Anti TTG negative	Gray Zone
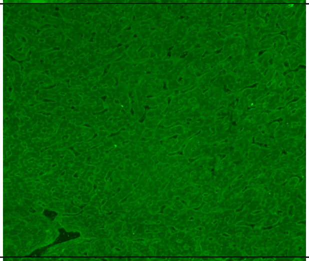	21	Positive	Near to negative but slight positivity in Anti TTG	Gray Zone
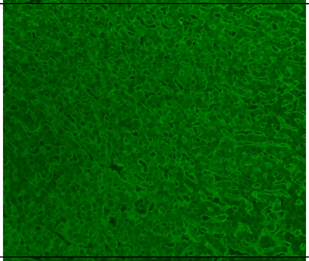	4,7	Negative	Near to +1 positive, but Anti TTG negative	Gray Zone
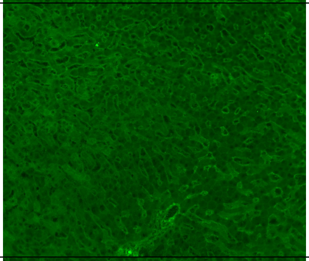	10,3	Negative	Near to +1 positive, but Anti TTG negative	Gray Zone
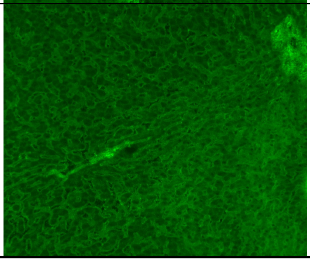	<2	Negative	Near to +1 positive, but Anti TTG negative	Gray Zone

Continued on next page

Image	Anti TTG IgA ELISA ($\succ$20 RU/mL positive)	Anti TTG result	Experts' Final Consideration	ML Prediction
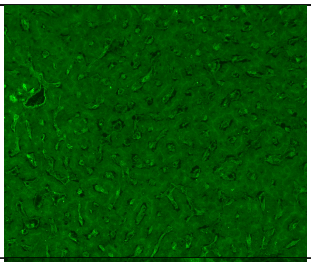	23,7	Positive	Near to negative but slight positivity in Anti TTG	Gray Zone
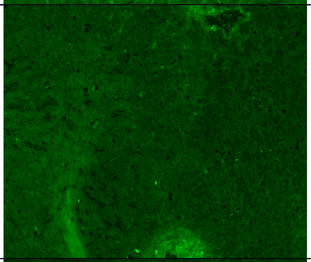	<2	Negative	Near to +1 positive, but Anti TTG negative	Gray Zone
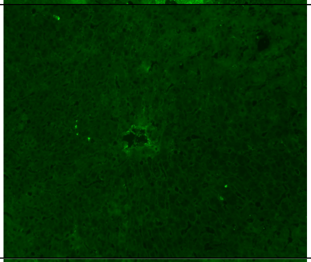	<2	Negative	Near to +1 positive, but Anti TTG negative	Gray Zone
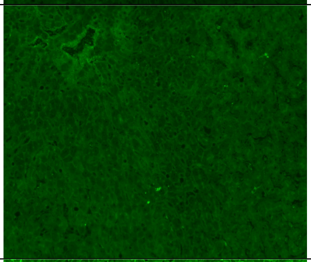	<2	Negative	Near to +1 positive, but Anti TTG negative	Gray Zone
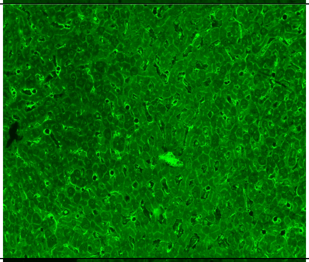	21	Positive	Near to negative but slight positivity in Anti TTG	Gray Zone
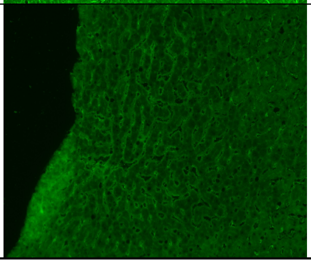	4,7	Negative	Near to +1 positive, but Anti TTG negative	Gray Zone

Continued on next page

Image	Anti TTG IgA ELISA ($\succ$20 RU/mL positive)	Anti TTG result	Experts' Final Consideration	ML Prediction
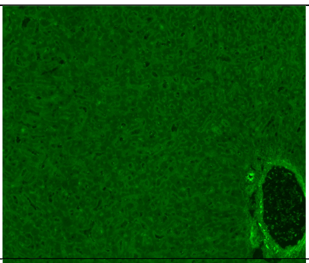	10,3	Negative	Near to +1 positive, but Anti TTG negative	Gray Zone
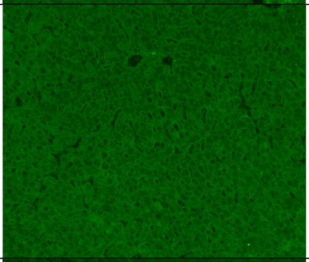	22,4	Positive	Near to negative but slight positivity in Anti TTG	Gray Zone
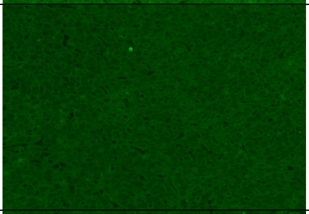	8,3	Negative	Near to +1 positive, but Anti TTG negative	Gray Zone
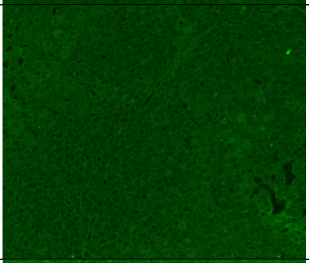	7,2	Negative	Near to +1 positive, but Anti TTG negative	Gray Zone
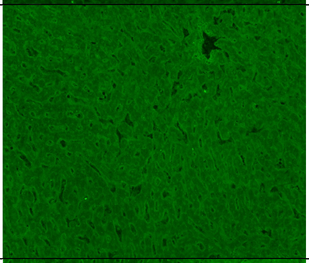	18,9	Negative	Near to +1 positive, but Anti TTG negative	Gray Zone
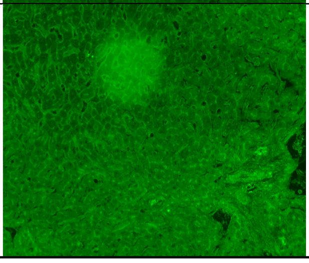	7,1	Negative	Near to +1 positive, but Anti TTG negative	Gray Zone

Continued on next page

**Image**	**Anti TTG IgA ELISA ($\succ$20 RU/mL positive)**	**Anti TTG result**	**Experts' Final Consideration**	**ML Prediction**
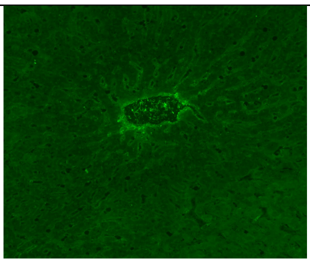	<2	Negative	Heterogenous staining, neither +1 positive nor negative.	Gray Zone
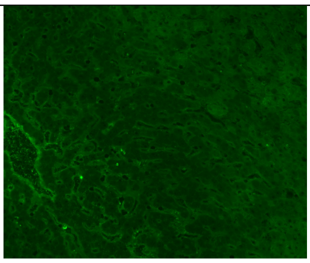	32,4	Positive	Heterogenous staining, neither +1 positive nor negative.	Gray Zone
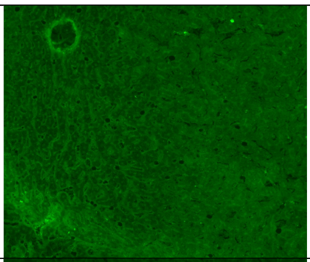	<2	Negative	Near to +1 positive, but Anti TTG negative	Gray Zone
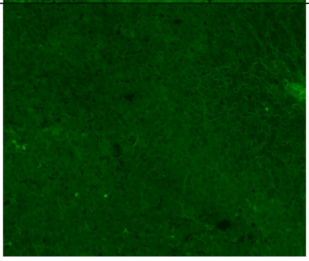	30,7	Positive	Heterogenous staining, neither +1 positive nor negative.	Gray Zone
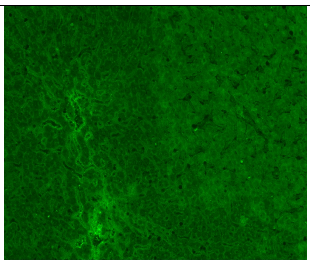	57,3	Positive	Heterogenous staining, neither +1 positive nor negative.	Gray Zone
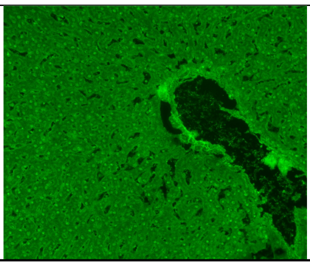	11,3	Negative	Near to +1 positive, but Anti TTG negative	Negative

Continued on next page

**Image**	**Anti TTG IgA ELISA ($\succ$20 RU/mL positive)**	**Anti TTG result**	**Experts' Final Consideration**	**ML Prediction**
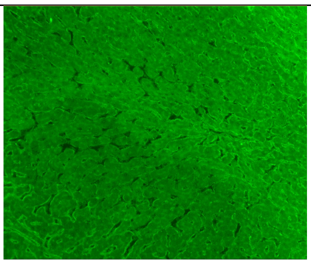	3,6	Negative	Heterogenous staining, neither +1 positive nor negative.	Gray Zone
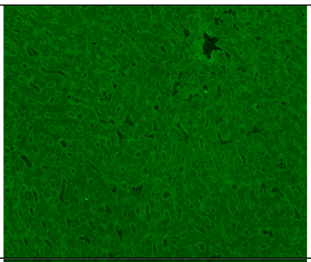	131,1	Positive	Near to negative but has strong Anti TTG positivity	Gray Zone
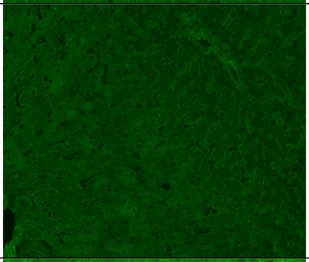	31,3	Positive	Near to negative but slight positivity in Anti TTG	Gray Zone
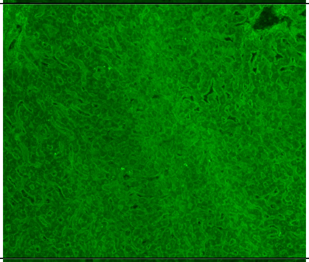	10,3	Negative	Near to +1 positive, but Anti TTG negative	Gray Zone
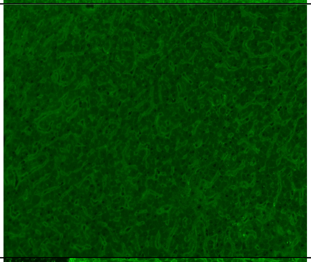	97,5	Positive	Near to negative but has strong Anti TTG positivity	Gray Zone
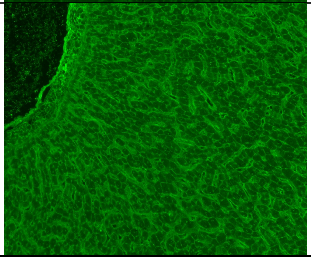	2,5	Negative	Near to +1 positive, but Anti TTG negative	Gray Zone

Continued on next page

**Image**	**Anti TTG IgA ELISA ($\succ$20 RU/mL positive)**	**Anti TTG result**	**Experts' Final Consideration**	**ML Prediction**
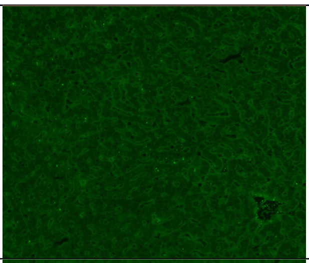	18	Negative	Near to +1 positive, but Anti TTG negative	Gray Zone
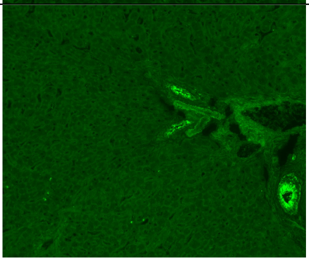	3,4	Negative	Heterogenous staining, neither +1 positive nor negative.	Negative
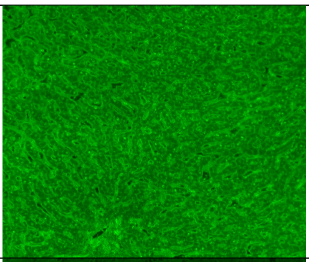	7,1	Negative	Near to +1 positive, but Anti TTG negative	Gray Zone
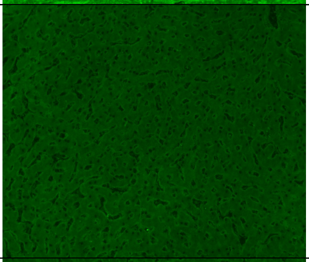	2,4	Negative	Near to +1 positive, but Anti TTG negative	Gray Zone
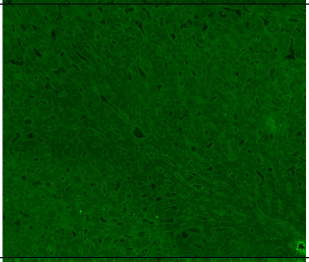	30,1	Positive	Near to negative but slight positivity in Anti TTG	Gray Zone
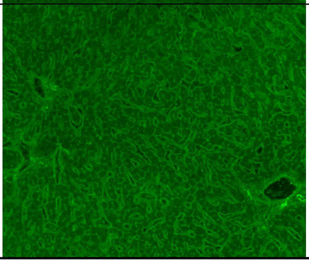	<2	Negative	Near to +1 positive, but Anti TTG negative	Weak Positive

Continued on next page

Image	Anti TTG IgA ELISA ($\succ$20 RU/mL positive)	Anti TTG result	Experts' Final Consideration	ML Prediction
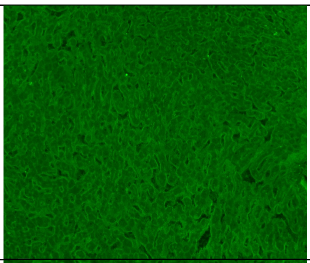	5,9	Negative	Near to +1 positive, but Anti TTG negative	Gray Zone
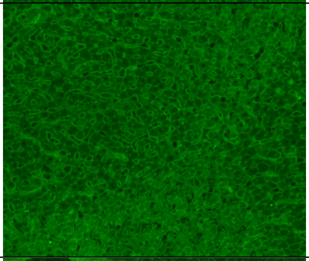	<2	Negative	Near to +1 positive, but Anti TTG negative	Gray Zone
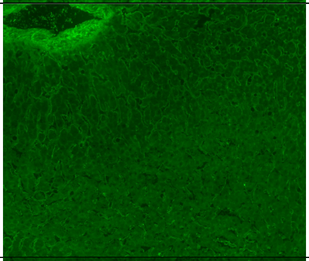	9,3	Negative	Near to +1 positive, but Anti TTG negative	Gray Zone
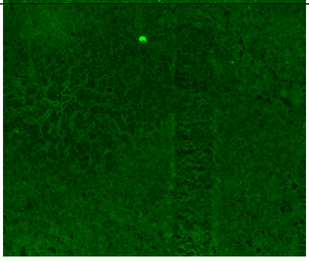	47,6	Positive	Heterogenous staining, neither +1 positive nor negative.	Gray Zone
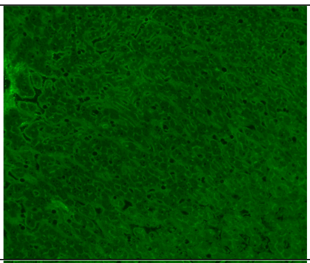	<2	Negative	Near to +1 positive, but Anti TTG negative	Gray Zone
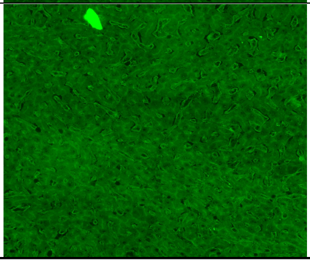	<2	Negative	Near to +1 positive, but Anti TTG negative	Gray Zone

Continued on next page

**Image**	**Anti TTG IgA ELISA ($\succ$20 RU/mL positive)**	**Anti TTG result**	**Experts' Final Consideration**	**ML Prediction**
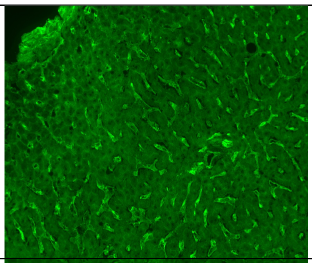	4,8	Negative	Near to +1 positive, but Anti TTG negative	Gray Zone
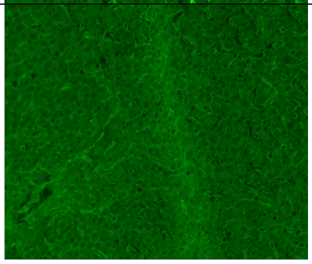	7,4	Negative	Heterogenous staining, neither +1 positive nor negative.	Gray Zone
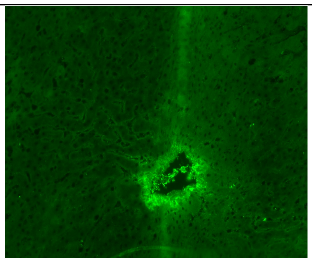	<2	Negative	Heterogeneous staining, neither +1 positive nor negative.	Gray Zone
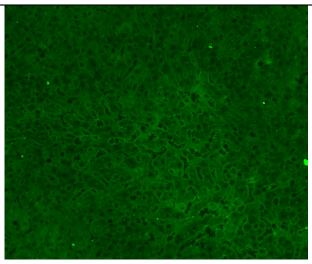	12,3	Negative	Heterogeneous staining, neither +1 positive nor negative.	Gray Zone

Table 2: TTG Analysis Results with Grayzone Images
